# Quadriceps muscle electromyography activity during physical activities and resistance exercise modes in younger and older adults

**DOI:** 10.1016/j.exger.2020.110965

**Published:** 2020-07-15

**Authors:** Ryan N. Marshall, Paul T. Morgan, Eduardo Martinez-Valdes, Leigh Breen

**Affiliations:** a School of Sport, Exercise and Rehabilitation Sciences, University of Birmingham, United Kingdom; b MRC-Versus Arthritis Centre for Musculoskeletal Ageing Research, University of Birmingham, United Kingdom; c NIHR, Birmingham Biomedical Research Centre, Birmingham, United Kingdom; d Centre of Precision Rehabilitation for Spinal Pain (CPR Spine), School of Sport, Exercise and Rehabilitation Sciences, College of Life and Environmental Sciences, University of Birmingham, United Kingdom

**Keywords:** Ageing, Muscle, Electromyography, Resistance exercise training

## Abstract

**Background:**

Understanding the root cause of the age-related impairment in muscle adaptive remodelling with resistance exercise training (RET) and developing pragmatic and accessible resistance exercise for older adults, are essential research directives.

**Methods:**

We sought to determine whether indices of quadriceps muscle EMG activity in response to different modes of RET and activities of daily living (ADL), differed between 15 healthy younger (25 ± 3 years) and 15 older (70 ± 5 years) adults. On four separate days, participants completed a maximal voluntary contraction (MVC) of the knee extensors, followed by a 15 m walking task, stair climbing task (i.e. ADL) and lower-limb RET through body-weight squats (BW-RET) and seated knee extensions on a machine (MN-RET) or via elastic bands (EB-RET). Surface quadriceps electromyography (EMG) was measured throughout all tasks to provide indirect estimates of changes in muscle activity.

**Results:**

MVC was significantly greater in young vs. older adults (Young: 256 ± 72 vs. Old: 137 ± 48 N·m, P < 0.001). EMG activity during all exercise tasks was significantly higher in older vs. younger adults when expressed relative to maximal EMG achieved during MVC (P < 0.01, *for all*). In addition, relative quadriceps muscle EMG activity was significantly greater in EB-RET (Young: 20.3 ± 8.7 vs. Old: 37.0 ± 10.7%) and MN-RET (Young: 22.9 ± 10.3, vs. Old: 37.8 ± 10.8%) compared with BW-RET (Young: 8.6 ± 2.9 vs. Old: 27.0 ± 9.3%), in young and older adults (P < 0.001). However, there was no significant difference in quadriceps EMG between EB-RET and MN-RET (P > 0.05).

**Conclusions:**

In conclusion, relative quadriceps muscle EMG activity was higher across a range of activities/exercise modes in older vs. younger adults. The similar quadriceps muscle EMG activity between EB-RET and MN-RET provides a platform for detailed investigation of the neuromuscular and muscle metabolic responses to such pragmatic forms of RET to strengthen the evidence-base for this mode of RET as a potential countermeasure to sarcopenia.

## Highlights

• Ageing results in severe declines of neuromuscular function and strength.
• Activities of daily living require greater relative muscle activity in older compared with younger adults.
• Elastic band resistance exercise produces comparable quadriceps muscle EMG activity compared with traditional machine-based exercise, irrespective of age.
• Elastic band resistance exercise *may* represent a novel exercise modality in facilitating muscular adaptation in older adults.
• Bodyweight resistance exercise evokes lower muscle activity compared with elastic based and machine-based exercise, however, may still provide a potent resistance exercise stimulus for older adults.

## 1 Introduction

Ageing is associated with a precipitous loss of skeletal muscle strength, quantity/quality and functional capacity (termed sarcopenia) (Cruz-Jentoft et al., 2020; Rosenberg, 1997). Sarcopenia progresses at a rate of ~0.7–1.2%/year after the 5th decade of life (Mitchell et al., 2012), and is associated with a loss of independence (dos Santos et al., 2017) and increased morbidity and mortality risk (Brown et al., 2016). The prevalence of sarcopenia within the UK, in community-dwelling older adults (>65 years), has been reported to be >12.5% (Patel et al., 2013), rising to >20% in those aged >85 years (Dodds et al., 2017). Alarmingly, >78% of older adults admitted to primary care and inpatient facilities are diagnosed with this condition (Rubio-Maicas et al., 2014). Ultimately, sarcopenia presents a considerable burden on healthcare resources (Goates et al., 2019) that will continue to rise as prevalence increases (Ethgen et al., 2017).

Evidence from randomised control trials (Leenders et al., 2012; Snijders et al., 2019; Verdijk et al., 2009), systematic reviews and meta-analyses (Peterson et al., 2010; Peterson et al., 2011) demonstrates that regular, progressive resistance exercise training (RET) can effectively attenuate/counteract sarcopenia and alleviate many of its adverse health consequences. However, the muscle adaptive remodelling response to RET may be compromised in older age (Greig et al., 2011). Specifically, there is strong evidence that the muscle protein synthetic (MPS) response to RET is blunted in older, compared with younger, adults (Brook et al., 2016). This is important as RET-induced MPS stimulation is a key determinant of the overall net protein balance/accretion for muscle hypertrophy (Morton et al., 2018). However, the underlying cause of this muscle anabolic resistance in older age is unclear and, therefore, warrants further investigation in order to maximize the health benefits of RET in older adults.

Contraction-induced MPS stimulation is dependent on a number of highly coordinated neurophysiological and muscle metabolic processes (Wilkinson et al., 2018). Motor unit recruitment and maximal fibre activation are thought to be crucial for RET-induced MPS stimulation (Burd et al., 2012; Burd et al., 2010). Indeed, the compromised properties of the ageing neuromuscular system are suggested as key factors in the loss of muscle fibre size, fibre number and motor performance (Piasecki et al., 2016) and, thus, could be implicated in the attenuated MPS response to RET. To date, very few studies have investigated how RET-induced neurophysiological responses are altered by ageing, partly due to the technical challenges of measuring these parameters in vivo under exercise conditions (Piasecki et al., 2018a). Surface electromyography (EMG) is an accessible, non-invasive tool that can be used to measure the transmembrane current of muscle fibres (or ‘muscle activity’), that, when applied in such a way to overcome the well-described shortcomings of this method (Vigotsky et al., 2018), can be useful for gaining insight into neuromuscular responses to ageing, disease and exercise (Vigotsky et al., 2018). As such, EMG application may enhance our understanding of potential age-related changes in RET-induced muscle activation.

Despite the well-established effects of RET, adherence and compliance to such gym-based RET in an unsupervised environment is underwhelming (Burton et al., 2017). This may, in part, be due to cost and aversion to commercial gym facilities in older adults (Timmons et al., 2020). As such, there is a need to develop pragmatic and accessible RET interventions that can effectively support the maintenance or, indeed, enhancement of muscle mass, strength and function in older age (Timmons et al., 2020). Elastic resistance bands (EB) and body-weight (BW) lifting are proficient methods of RET, capable of increasing muscle mass, strength and functional capacity in community-dwelling (Fujita et al., 2016; Krause et al., 2019), institutionalized (Fujita et al., 2019; Furtado et al., 2020), sarcopenic-obese older adults (Liao et al., 2018), and older adults with cognitive impairment (Chupel et al., 2017). Compared with traditional machine-based (MN) RET, EB have also been shown to generate an ‘ascending’ (or ‘linear variable’) resistance training load, providing an increasing tensile load due to the stretch properties of the EB (Fuentes et al., 2019). In addition, EB-RET demonstrates similar EMG activity when compared with machine and free-weight alternatives during multi-joint movements in younger adults (Calatayud et al., 2014; Calatayud et al., 2015; Iversen et al., 2017; Jakobsen et al., 2013), and in clinical rehabilitation patients (Jakobsen et al., 2019; Vinstrup et al., 2017). Given the dearth of work in this area, there is a need for improving understanding of the neuromuscular and muscle metabolic responses to EB-RET and BW-RET in older adults, particularly in comparison with traditional machine-based RET and the response in younger adults. Addressing these important questions will enhance the evidence-base for RET recommendations and prescription in older adults.

The primary purpose of the present study was, therefore, to assess quadriceps muscle EMG activity using textile embedded EMG shorts during BW-RET, EB-RET and MN-RET and activities of daily living (ADL) in younger and older adults to understand the effects of ageing and contractile mode on muscle activity. We hypothesized that BW-RET would result in lower relative EMG activity compared to EB-RET and MN-RET in younger adults, however, that there would be no difference between RET tasks in older adults. Moreover, EB-RET would elicit similar quadriceps muscle EMG activity to that observed with traditional MN-RET, irrespective of age. Subsequently, the secondary purpose of the study was to determine the effects of ADL (activities of daily living: walking and stair climbing) on quadriceps muscle EMG activity in younger and older adults. We hypothesized that quadriceps muscle EMG activity, relative to that achieved during a maximal voluntary contraction (MVC), would be greater in older vs. younger adults across all RET modalities and ADL.

## 2 Materials and methods

### 2.1 Participants

Fifteen healthy younger (25 ± 3 years, 8 men, 7 women, BMI: 24.3 ± 3.0 kg/m^2^) and fifteen older (70 ± 5 years, 8 men, 7 women, BMI: 23.6 ± 3.4 kg/m^2^) volunteers participated in this study. All study participants were recreationally active, and defined as healthy, as determined via a general health screening and medical questionnaire. Briefly, inclusion criteria included being habitually or recreationally active, non-smoker, non-diabetic and normotensive (<140/90). Exclusion criteria included; a highly sedentary lifestyle (<5000 steps per day), hypertensive (>140/90), the consumption of non-steroidal anti-inflammatory drugs, and the consumption of caffeine ~12 h prior to an experimental visit. This study was conducted in accordance with the declaration of Helsinki, and ethical approval was obtained from the University of Birmingham (ERN_19–0220). After being informed of the experimental procedures and associated risks, all participants provided written informed consent prior to engaging in the study.

### 2.2 Experimental design

Participants visited the laboratory on four separate occasions over a 2-week period, with all tests separated by at least 48 h and conducted at a similar time of day (± 90 min) to limit potential changes in quadriceps strength and to account for diurnal variations in neuromuscular excitability (Souissi et al., 2002). Using a within-subject design and following familiarization and strength testing (see ‘Familiarization and Strength Testing’), participants visited the lab on three separate occasions to perform BW-RET, EB-RET and MN-RET (see ‘Experimental Trials’). Prior to each experimental visit, participants consumed their habitual diet and were instructed not to participate in any rigorous physical activity or consume alcohol ~48 h and ~ 24 h, respectively, prior to the visit. Briefly, on each of the trial days, participants wore textile surface EMG shorts (see ‘Surface Electromyography’) to provide an indirect measure of muscle activity during each exercise task. Following a standardised warm-up, participants performed six sets of twelve repetitions at ~70% of their 1 repetition maximum (1-RM), with a 120 s intra-set recovery period. Immediately after each set, perceived exertion (RPE) was determined using a modified Borg scale (Buckley and Borg, 2011). This method of assessing perceived exertion during resistance exercises is comparable to other RPE scales currently validated for monitoring intensity during resistance exercises performed with elastic bands in older populations (Colado et al., 2020; Colado et al., 2018).

### 2.3 Familiarization and strength testing

The first laboratory visit was used to assess body composition, as determined via bioelectrical impedance analysis (mBCA 525, SECA, Hamburg, Germany), incorporating assessments of fat mass (FM), body fat percentage (BF%), fat-free mass (FFM) and skeletal muscle mass (SMM). BIA has previously been observed to be a reliable and valid measure of body composition (Bosy-Westphal et al., 2017). In addition, MVC of the knee extensors was assessed using an isokinetic dynamometer (KinCom 125AP, KinCom, USA) for evaluation of quadriceps isometric muscle strength and maximal EMG activity of both limbs. Briefly, participants were seated with the hip and knee joints at relative angles of 90° and 110°, respectively, with the tested limb, chest and hips, stabilised (Häkkinen et al., 2000) and adjusted so that the axis of rotation of the lever arm was in line with the lateral epicondyle of the femur. All participants completed 3 × 3 s MVC of the knee extensors with each contraction separated by a 60 s passive recovery period. Participants were given standardised verbal encouragement for the duration of each 3 s contraction. All participants then completed 3 × 15 m walk at their regular walking pace, followed by the completion of the descent and ascent of a flight of stairs (12 steps, 30 cm each in height) to determine the effects on quadriceps muscle EMG.

Participants were then familiarised to the exercise tasks for the experimental trials, as described below, which included the assessment of a 12-repetition maximum (12-RM) for EB-RET and MN-RET. Subsequently, participants performed three experimental trials: (Aboodarda et al., 2016) BW-RET, (Arentson-Lantz et al., 2019) EB-RET and (Baggen et al., 2019) MN-RET. MN-RET 12-RM was determined using a knee extension strength training machine (Elite Series Leg Extension/Curl, Fitness Warehouse Ltd., Preston, Lancashire, UK), starting with a self-selected load estimated to be ~50% of the participants' 1-RM. Following the completion of a standardised warm-up and 12 successful repetitions, the load was increased by ~5–10 kg increments until the participant reached the desired load representing their 12-RM (~70% 1-RM). The ‘desired’ load was estimated based upon participants' rating of perceived exertion (RPE), between 7 and 8 (Helms et al., 2016). For EB-RET, the 12-RM load was assessed using an elastic resistance band (PeakSupps, Bridgend, UK) with loads ranging from ‘light’ to ‘very heavy’ (6 kg, 12 kg, 20 kg and 28 kg at 100% elongation of manufacturer guidelines). Using the machine-based variation as a guide, the participants commenced knee extension exercise testing with a band representing ~50% of their 1-RM. Following two initial warm-up sets, the load was increased via the increased elongation of the band in 20 cm increments and/or an increase in band resistance (i.e. band colour) until the participant reached a load representing their 12-RM, as described above. Participants were instructed to perform each repetition over a total of 3 s (eccentric phase: ~1.5 s and concentric phase: ~1.5 s), timed via a visual prompt to ‘push’ and ‘relax’, accompanied by the same verbal instructions from the experimenter. The starting position of EB-RET was subject to an initial pre-stretch of the band, providing a load at the start of each contraction and was, therefore, comparable to the starting position of MN-RET.

### 2.4 Experimental trials

Visits 2–4 were completed in a randomised order. For BW-RET (chair squats), participants were required to stand shoulder width apart in front of a standardised chair (height: 43 cm) and squat down to lightly touch the chair before ascending to a standing position with each repetition being completed within ~3 s and with a 120 s intra-set recovery period. The second and third experimental RET conditions required participants to undertake a single bout of EB and MN knee extension resistance exercise, respectively. For EB-RET and MN-RET, the trials started with a standardised warm-up routine consisting of two sets of twelve repetitions at 50% and 75% of their 12-RM, respectively. Participants then undertook a bout of knee extension exercise comprising of the same training volume, intra-set recovery and exercise cadence as BW-RET at their 12-RM. Figs. 4 and 5 provide a visual example of a participant undertaking the ADL and RET tasks, respectively, and are available as an online supplement.

### 2.5 Surface electromyography

EMG was used to quantify the electric potential of quadriceps and referred to as ‘quadriceps muscle EMG activity’ throughout (Vigotsky et al., 2018). Specifically, surface EMG was recorded from the *m.*vastus lateralis and *m.*vastus medialis of the right and left leg quadriceps, using textile shorts made of polyamide (71%) and elastane (29%) with bipolar electrodes integrated into the fabric (Mbody, Myontec Ltd., Kuopio, Finland). The electrodes connected wirelessly to an electronics module attached to the front of the shorts (MCell, Myontec Ltd., Kuopio, Finland), as previous described (Tikkanen et al., 2013). The relative position of the two quadriceps electrodes were such that they were located over the distal portion of the quadriceps muscle, superficial to the *m.*vastus lateralis and *m.*vastus medialis, as previously described (Tikkanen et al., 2013). The sizes of the electrodes are 2.5 cm × 9-14 cm depending on the size of the shorts. The reference electrodes are placed longitudinally over the tractus iliotibialis on either side of the shorts. The MCell contains the signal amplifiers, microprocessor and data memory. A range of sizes were used to ensure close proximity of the electrodes to the skin and to maximize the sensitivity of the EMG signal. Where appropriate, compression sleeves were used to ensure sufficient electrode contact with the skin superficial to the muscles of interest. Furthermore, the skin area underneath each EMG electrode was sprayed with water to optimise conductivity between the skin and electrodes. This method of EMG assessment has previously been established to be a valid and feasible method of assessing rectified values of muscle EMG activity during day-to-day functional exercise tasks (Brook et al., 2016; Colado and Triplett, 2008). Fig. 6 provides an annotated image of the textile EMG shorts used in the current study and is available as an online supplement.

### 2.6 Data analysis

The EMG signals were pre-amplified (1000 Hz) and then band-pass filtered (2nd order) with cut-off frequencies of ~40–300 Hz. EMG data were recorded continuously and digitised synchronously with 12-bit resolution via an A/D converter. The 1000 Hz signal was first rectified in an automated fashion and then averaged over non-overlapping 100 ms intervals, similar to the root mean square method. The averaged data was stored in the memory of the MCell at 25 Hz, from which the data was extracted and analysed using customised software (Muscle Monitor, Myontec Ltd., Kuopio, Finland) (Supplementary Fig. 7.). All EMG data was visually assessed and corrected for artefacts, as previously described (Tikkanen et al., 2013). Signal noise was determined via a test repetition during the warm-up sets prior to the main bout of exercise. To obtain an average rectified value of EMG amplitude, the average EMG signal amplitude was taken from the onset of the exercise task (or ‘set’) until task end, for both young and older groups. Following completion of each individual exercise session (i.e. walking, stair climb, BW-RET, EB-RET and MN-RET), the mean EMG amplitude was determined (i.e. the mean EMG amplitude of all 6 sets of 12 repetitions during RET) and presented relative to the maximal EMG amplitude achieved from a 1-s peak period during MVC.

### 2.7 Statistical analyses

A power analysis with an error = 0.05, power = 0.95, and effect size = 6.57, was performed using G x Power 3.1 analysis software (Heinrich Hein University, Duesseldorf, Germany) based on the differences in walking EMG activity between younger and older adults (Hortobágyi et al., 2011). However, an additional power analysis with an error = 0.05, power = 0.95, and effect size = 1.32, was performed based on the differences in RMS EMG activity during knee extension contractions between younger and older adults (Bazzucchi et al., 2004). This produced a minimum sample size of 14 participants. A total of 15 per group were recruited to account for differences between measurement methods, age and activities of daily living and RET modes to provide a more detailed characterisation of our outcome measures. Independent samples *t*-tests were used to assess differences in components of body composition (i.e., body fat %, fat mass, fat-free mass and skeletal muscle mass) and EMG amplitude (i.e., muscle activity) between groups (i.e., young and old). In addition, differences in maximal (i.e., knee extensor MVC torque) and sub-maximal (i.e., EB 12-RM, MN 12-RM) strength were also assessed via independent samples *t*-tests. Two-way mixed-model repeated-measures ANOVAs (group x resistance exercise modality or ADL task) were used to compare quadriceps muscle EMG activity and perceived exertion (i.e., RPE) between resistance exercise modes (i.e., BW-RET, EB-RET and MN-RET) and between ADL (i.e., 15 m walk, stair climb descent and ascent). Where the ANOVA revealed a significant interaction effect, post-hoc *t-*tests were completed using Tukey's HSD. Normal distribution was assessed using the Shapiro-Wilk test. For calculation of effect size, partial eta squared (η^2^) was used for omnibus tests and Cohen's *d* was used for *t*-tests and post-hoc comparisons. All statistical tests were performed on relative and raw absolute data. However, unless otherwise stated, relative data is presented for all variables. Where sphericity was violated, a Greenhouse-Geisser correction factor was applied. For all tests, results were considered statistically significant when P < 0.05. Data are presented as mean ± standard deviation, unless otherwise stated. All statistical analyses were conducted using IBM SPSS Statistics version 25 (IBM SPSS inc. Chicago, Illinois, USA).

## 3 Results

### 3.1 Body composition

Components of body composition for both groups can be seen in Table 1. No differences were observed between groups for FM (P = 0.46, *d* = 0.27) or BF% (P = 0.17, *d* = 0.51). However, whilst there was no significant difference between groups for FFM, there was a strong effect size, implying a higher FFM in the young group (P = 0.06, *d* = 0.71). In addition, SMM was greater in the young compared with the old group (P = 0.01, *d* = 0.91). The coefficient of variation of BIA in our lab measurements of body composition for each group were; FM (Young, 0.9 ± 0.2% vs Old, 0.4 ± 0.4%), BF% (Young, 0.8 ± 1.2% vs Old, 0.5 ± 0.4%), FFM (Young, 0.2 ± 0.4% vs Old, 0.1 ± 0.1%), SMM (Young, 0.3 ± 0.4% vs Old, 0.2 ± 0.2%), respectively, with no differences observed between groups (P > 0.3 for all).

**Table 1:** Participants anthropometric, body composition and strength characteristics in young and old adults.

	Young (N = 15)	Old (N = 15)	P value	Effect size
Anthropometrics				
Age (years)	25.1 ± 3.0	70.8 ± 5.4	<0.001‡	–
Height (cm)	171.8 ± 7.0	167.9 ± 8.5	0.18	–
Body mass (kg)	72.3 ± 12.2	67.0 ± 12.3	0.25	–
BMI (kg^.^m^−2^)	24.4 ± 3.0	23.6 ± 3.5	0.54	–

Knee extensor maximal and submaximal strength				
MVC (N·m)	256 ± 72	137 ± 48	<0.001‡	2.01
KET/BM (N^.^m^.^kg^−1^)	3.5 ± 0.7	2.1 ± 0.6	<0.001‡	2.16
EB 12-RM (kg)	17 ± 3	12 ± 3	0.002⁎	1.25
MN 12-RM (kg)	17 ± 7	10 ± 4	<0.001‡	1.54

Body composition				
Whole body FFM (kg)	55.6 ± 10.9	43.4 ± 9.4	0.06	0.71
Whole body FM (kg)	16.7 ± 5.1	20.9 ± 14.2	0.47	0.27
Body fat (%)	23.2 ± 6.6	27.3 ± 9.6	0.17	0.51
Skeletal muscle mass (kg)	27.4 ± 6.3	21.7 ± 5.2	0.01†	0.91

BMI, Body Mass Index; MVC, Maximal Voluntary Contraction; KET/BM, Knee Extension Torque Relative to Body Mass; EB, Elastic Band, MN, Machine; FFM, Fat Free Mass; FM, Fat Mass; FFM = fat-free mass; FM = fat mass. Data presented as mean ± SD.

⁎ Significantly different from young (P < 0.05).

† Significantly different from young (P < 0.01).

‡ Significantly different from young (P < 0.001).

### 3.2 Knee extensor strength

**Fig. 1:**
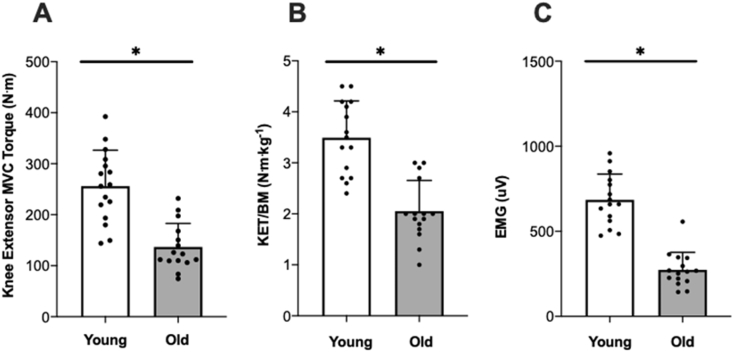
Maximal knee extensor torque and peak EMG activity. Group mean ± standard deviation for (A) Maximal quadriceps knee extension torque (N^.^m), (B) maximal quadriceps knee extension torque relative to bodyweight (N^.^m^.^kg^−1^); and (C) peak electromyography (EMG) activity (uV) achieved during a 90^o^ isometric knee extensor contraction for young (white bars) and old (grey bars) adults. * Indicate significant (P < 0.05) differences between groups. MVC, Maximal voluntary contraction; KET, Knee-extension torque; BM, Body mass.

Data for maximal and sub-maximal knee extensor strength are seen in Table 1. Knee extensor MVC torque was ~46% lower in the old compared with the young group (P < 0.001, *d* = 2.01, Fig. 1). In addition, knee extension torque (KET), relative to body mass (N^.^m/kg), was ~66% greater in the young group (P < 0.001, *d* = 2.16, Fig. 1). 12-RM strength was higher in the young group for EB (P = 0.002, *d* = 1.25) and MN (Young, P < 0.001, *d* = 1.54), with a 40% and 27% difference in 12-RM strength, respectively, between groups.

### 3.3 Muscle EMG activity

**Fig. 2:**
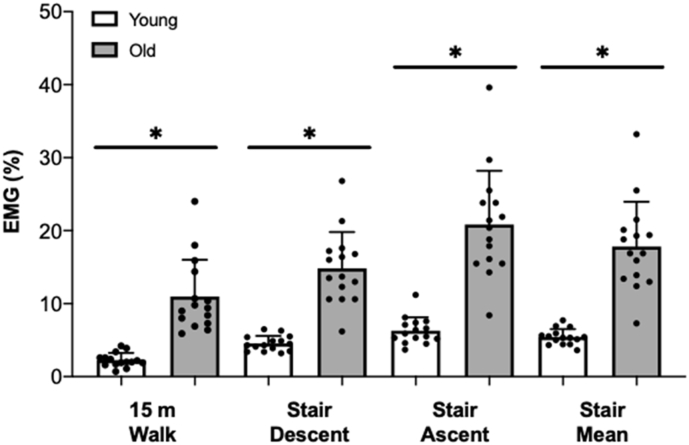
Quadriceps muscle EMG activity during activities of daily living. Peak muscle EMG activity of the quadriceps during activities of daily living (walking, stair descent, stair ascent and stair mean) in young (white bars) and old (grey bars) adults, with values presented as means ± standard deviation. * Indicate significant (P < 0.01) differences in muscle EMG activity (EMG, %) between groups. EMG, Electromyography.

**Fig. 3:**
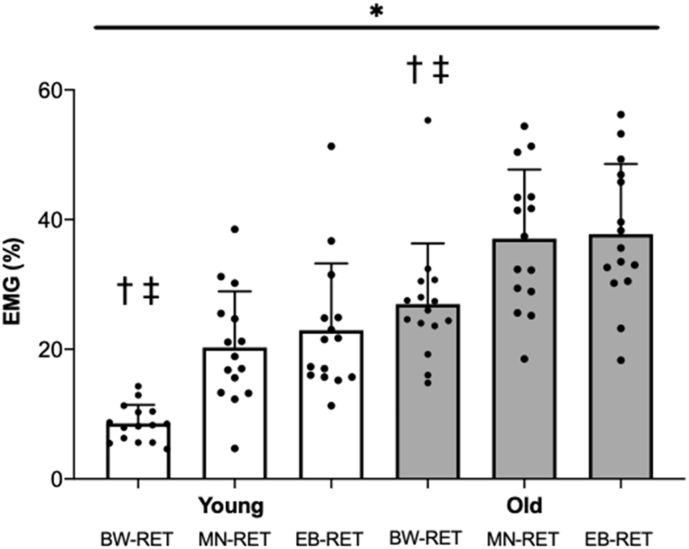
Quadriceps muscle EMG activity during bodyweight, elastic and machine resistance exercise. EMG activity of the quadriceps during, bodyweight (BW-RET), elastic band (EB-RET) and machine (MN-RET) resistance exercise training modalities in young (white bars) and old (grey bars) adults, with values presented as means ± standard deviation. * Indicates significant (P < 0.001) differences in muscle activity between groups; ^†^ significantly lower than MN-RET (P < 0.001); ^‡^ significantly lower than EB-RET (P < 0.001).

Maximal quadriceps muscle EMG activity, as measured during MVC, was higher in the young compared with the old group (P < 0.001, *d* = 3.23, Table 2, Fig. 1), with older adults exhibiting a ~ 60% difference in maximal quadriceps muscle EMG activity compared with their younger counterparts. We also observed significant main effects of group (i.e., old vs. young) and resistance exercise modality (i.e., BW-RET, EB-RET, MN-RET) on muscle activity (Table 2, Fig. 3, *both* P < 0.001, *both* η^2^ > 0.55). Similarly, we also observed significant main effects of group and ADL task on muscle activity (Table 2, Fig. 2, *both* P < 0.01, *both* η^2^ > 0.62). Specifically, during a 15 m walk, we observed a > 3-fold difference in relative quadriceps muscle EMG activity in the older compared with younger adults (Young: 2.3 ± 1.0% vs. Old: 11.0 ± 5.0%, P < 0.001, *d* = 2.91, Table 2, Fig. 2). In addition, older adults displayed a > 2-fold difference in quadriceps muscle EMG activity compared with their younger counterparts during the descent (Young: 4.5 ± 1.0% vs. Old: 14.8 ± 5.0%, P < 0.001, *d* = 3.12, Table 2, Fig. 2), ascent (Young: 6.3 ± 1.8% vs. Old: 20.8 ± 7.4%, P < 0.001, *d* = 3.43, Table 2, Fig. 2), and mean (ascent and decent) of the stair climb (Young: 5.4 ± 1.1% vs. Old: 17.8 ± 6.1%, P < 0.001, *d* = 3.44, Table 2, Fig. 2). Post-hoc tests revealed significant differences in quadriceps muscle EMG activity between all ADL tasks (i.e., 15 m walk, ascent and descent), irrespective of the group (all P < 0.01, Table 2, Fig. 2).

**Table 2:** Electromyography amplitude and perceived exertion during activities of daily living and resistance exercise training modalities.

	Young			Old			P Value	ES (d)
	EMG (uV)	EMG (%)	RPE (Borg)	EMG (uV)	EMG (%)	RPE (Borg)		
MVC	685 ± 152	–	–	273 ± 103	–	–	<0.001⁎	3.23
15 m walk	15.8 ± 8.2	2.3 ± 1.0	–	28.0 ± 11.5	11.0 ± 5.0	–	<0.001⁎	2.91
Stair climb								
Descent	30.3 ± 6.4	4.5 ± 1.0	-	36.7 ± 6.3	14.8 ± 5.0	-	<0.001⁎	3.12
Assent	42.3 ± 12.2	6.3 ± 1.8		51.7 ± 11.4	20.8 ± 7.4	-	<0.001⁎	3.43
Mean	36.3 ± 7.4	5.4 ± 1.1		44.2 ± 8.6	17.8 ± 6.1	-	<0.001⁎	3.44
Bodyweight chair squat	56.4 ± 16.2	8.6 ± 2.9	3.5 ± 1.3	67.3 ± 12.4	27.0 ± 9.3	4.4 ± 1.7	<0.001⁎	3.02
Elastic knee extension	132.9 ± 52.5	20.3 ± 8.7	6.8 ± 0.8	95.4 ± 29.1	37.0 ± 10.7	7.2 ± 0.7	<0.001⁎	1.74
Machine knee extension	149.5 ± 54.8	22.9 ± 10.3	7.1 ± 1.0	95.3 ± 20.6	37.8 ± 10.8	6.7 ± 1.2	<0.001⁎	1.41

EMG, Electromyography; MVC, Maximal Voluntary Contraction; ES, effect size. Data presented as mean ± SD. EMG (%) is expressed relative to maximal EMG achieved during MVC.

⁎ Significantly different from young (P < 0.001).

During BW-RET, older adults exhibited ~3-fold greater quadriceps muscle EMG activity than that of younger adults (Young: 8.6 ± 2.9% vs. Old, 27.0 ± 9.3%, P < 0.001, *d* = 3.02, Table 2, Fig. 3). In addition, quadriceps muscle EMG activity during EB-RET (Young: 20.3 ± 8.7% vs. Old: 37.0 ± 10.7%) and MN-RET (Young: 22.9 ± 10.3%, vs. Old: 37.8 ± 10.8%) were greater in older compared with younger adults (Table 2, Fig. 3, both P < 0.001, *d* > 1.40). Whilst BW-RET, in older adults, was associated with higher muscle EMG activity when compared with younger adults (Older: 73.4 ± 19.3% vs. Young: 42.3 ± 14.4%, when expressed relative to EB-RET and MN-RET), post-hoc tests revealed differences in muscle EMG activity in both groups between BW-RET and EB-RET (P < 0.001, *d* = 0.90) and BW-RET and MN-RET (P < 0.001, *d* = 1.03). However, no differences were observed between EB-RET and MN-RET (P = 0.25, *d* = 0.13).

### 3.4 Perceived exertion

A significant main effect of RET modality was observed on RPE (P < 0.01, η^2^ = 0.76, Table 2). However, no differences were found between young and older adults, irrespective of the RET modality (P = 0.86, η^2^ = 0.13, Table 2). Specifically, no differences in RPE between EB-RET and MN-RET were observed in the young (EB: 6.8 ± 0.8, vs. MN: 7.1 ± 1.0, P = 0.40, *d* = 0.31, Table 2) or older group (EB: 7.1 ± 1.0, vs. MN: 6.7 ± 1.2, P = 0.23, *d* = 0.47, Table 2). However, BW-RET resulted in significantly lower RPE in both groups when compared with EB-RET and MN-RET (Young: 3.5 ± 1.3 vs. Old: 4.5 ± 1.8, P = 0.12, *d* = 0.59, Table 2).

## 4 Discussion

The aim of this study was to determine quadriceps muscle EMG activity in response to various RET modalities and ADL in healthy younger and older adults. In agreement with our primary hypothesis, we demonstrated EB-RET to elicit comparable quadriceps muscle EMG activity to that of MN-RET in both younger and older adults when exercise volume and intensity was matched. However, whilst, in older adults, BW-RET (i.e. chair squats) elicited ~27% of maximal quadriceps muscle EMG activity, this was significantly lower (~73%) than the quadriceps muscle EMG activity evoked by EB-RET and MN-RET. Finally, and in agreement with our secondary hypothesis, we observed significantly greater quadriceps muscle EMG activity during all RET modalities and ADL (walking and stair climbing) in older compared with younger adults when expressed relative to maximal muscle EMG. Taken together, these data suggest that relative muscular effort (of the quadriceps) required to complete RET and ADL is greater in older compared with younger adults, and that EB-RET, but not BW-RET, can modulate quadriceps muscle EMG activity to a similar extent to that of traditional MN-RET, in healthy younger and older adults. Finally, and as expected, quadriceps muscle strength was ~46% lower in older compared with younger adults and this was associated with a ~ 60% lower peak quadriceps muscle EMG, used as an indirect measure of maximal muscle EMG activity.

Our observation that EB-RET, but not BW-RET, elicited similar increases in quadriceps muscle EMG activity compared with traditional MN-RET in healthy younger and older adults is in line with previous findings (Brandt et al., 2013; Jakobsen et al., 2019). However, whilst our data suggest EB-RET and MN-RET elicit similar quadriceps muscle EMG activity, recent evidence suggests EB-RET may, instead, provoke a greater increase in muscle activity compared with leg extension (~11%) and leg press (~27%) machine-based exercise, respectively, as observed in patients during recovery from total knee arthroplasty (Jakobsen et al., 2019). Nevertheless, recent systematic reviews have shown no differences in voluntary muscle activity (Aboodarda et al., 2016) as well as in muscle strength and function (Lopes et al., 2019), between EB-RET and MN-RET in healthy populations. Indeed, longitudinal evidence regarding the use of EB-RET has shown significant improvements in body composition, strength and physical function in older, overweight adults following short-term training interventions (8–12 weeks) of a similar magnitude to that reported with MN-RET (Huang et al., 2017). This is consistent with MN-RET and EB-RET comparisons, overall observing similar increases in muscle strength and function in both healthy and sedentary females (Colado et al., 2010; Colado and Triplett, 2008). Nonetheless, collectively, the available data suggest EB-RET as a viable stimulus to support longer-term muscle remodelling in older adults, with implications for maintenance of strength, function and quality of life.

Unsurprisingly, BW-RET elicited a lower degree of quadriceps muscle EMG activity compared with EB-RET and MN-RET in younger and older adults. Nonetheless, BW-RET may still provide a potent RET stimulus for older adults, particularly as this method of RET induced a 10–16% greater EMG response (relative to maximal EMG) compared with all ADL (BW-RET: ~27% vs. Walking: ~11% vs. Stair Climbing: ~17%). Indeed, quadriceps muscle EMG activity during BW-RET, in older adults, represented ~73% of that observed during EB-RET and MN-RET, compared with ~42% in younger adults. Moreover, it is likely that prescribing BW-RET at an equivalent relative load to EB-MET and MN-RET (i.e. 12-RM), would lead to greater quadriceps EMG activity which, potentially, may translate to higher EMG activity across all muscles of the lower limb. Indeed, recent recommendations suggest bodyweight exercise to be performed to volitional fatigue (Mcleod et al., 2019). In addition, it is likely that BW-RET may be more beneficial for adults with lower relative levels of lean and concomitant higher levels of inert mass, contributing to a higher resistive load. BW-RET may, therefore, represent a feasible loading intervention to be completed as part of a home or community-based RET programme to increase muscle strength and function in older adults (Fujita et al., 2019).

Irrespective of the potential limitations, the potency of BW-RET as a contractile stimulus for muscle activation has been demonstrated in older adults (Jakobsen et al., 2019). Specifically, Jakobsen and colleagues recently reported bodyweight sit-to-stand exercise resulted in a ~ 14% and 8% greater increase in voluntary muscle activity of the quadriceps compared with both leg press and knee extension machine exercises, respectively (Jakobsen et al., 2019). In contrast, the present study did not achieve such high levels of quadriceps muscle EMG activity during BW-RET. This discrepancy is likely explained by differences in the study population. Specifically, the higher muscle EMG may be explained by atherogenic inhibition of the quadriceps muscles following knee surgery rehabilitation (Jakobsen et al., 2019). Indeed, our data are in agreement with Fujita and colleagues who revealed that, with increasing age, muscle activity substantially increases during a ‘sit-to-stand’ squat exercise (Young, ~14%; Middle-aged, ~25%; Older adults ~52%; Frail older adults ~72%) (Fujita et al., 2011). Furthermore, recent longitudinal evidence from the same group demonstrated marked improvements in muscle strength (>22%) following a sit-to-stand exercise regime in frail institutionalized older adults (Fujita et al., 2019). This, in combination with our data, suggests that BW-RET may provide a potent and pragmatic RET stimulus, particularly in older adults.

In younger adults, we reported quadriceps muscle EMG activity during a 15 m walk at ~2% relative to that achieved during MVC, which was significantly lower than quadriceps muscle EMG in older adults (~11%). These observations are comparable with observations in middle-aged adults (~10%) using similar EMG technology during ADL (Tikkanen et al., 2013). The differences in muscle EMG activity of older adults may be explained by lower relative lean mass, reduced muscular metabolic efficiency (i.e. increased consumption of ~7–20% more metabolic energy to walk a given distance compared to younger adults) (Hortobágyi et al., 2011), increased leg muscle coactivation (i.e. increased activation of agonist [quadriceps] and antagonist muscles [bicep femoris, gastrocnemius and tibialis anterior] during walking) (Hortobágyi and DeVita, 2000; Hortobágyi and DeVita, 2006; Hortobágyi et al., 2011) and impaired neuromuscular function (i.e. motor unit potential, compound muscle action potential) (Landers et al., 2001). This may be partially explained via the increase in muscle activation prior to foot contact when walking, which is accompanied by coactivation of antagonist muscles (Hortobágyi and DeVita, 1999). This occurrence is favourable in older adults to maintain joint stability during gait and stair climbing as a method to avoid potential falls (Hortobágyi et al., 2009). Nevertheless, in light of the relatively low muscle activation response to walking, relative to RET modes, it is likely that this mode of activity does not provide a sufficient stimulus in attenuating muscle loss with ageing. Furthermore, increased ambulation during disuse events (i.e. 2000 steps per/day on a hospital ward) was reported to have no effect on muscle mass in older adults (Arentson-Lantz et al., 2019). Indeed, maintaining or increasing habitual physical activity levels may be key to maintaining whole muscle neuromuscular characteristics as even brief periods of disuse and lower limb immobilisation have been shown to result in catastrophic declines in neuromuscular function (Campbell et al., 2019).

Quadriceps muscle EMG activity during the ascent of a stair climb task in older adults was ~20%, which is in agreement with previous research (Tikkanen et al., 2013). Interestingly, quadriceps muscle EMG activity was >3-fold greater in older compared with younger adults (~6%). As mentioned previously, the increase in muscle EMG activity may be due to reduced muscle metabolic and neuromuscular efficiency. Indeed, stair climbing is strongly associated with functional performance in community-dwelling and functionally impaired institutionalized older adults (Mair et al., 2019). In addition, previous research has observed significant improvements in cardiorespiratory fitness and muscle power (Bean et al., 2007) following a stair climbing programme, suggesting that this type of exercise task may provide an important stimulus for maintenance of muscle mass, strength and endurance in older adults (Hongu et al., 2019). Indeed, a twelve-week bodyweight stepping training intervention in older women was associated with significant improvements in thigh cross-sectional area volume (~2.8%) and isometric strength (~9–15%) (Baggen et al., 2019), similar to that observed following a high intensity (2 × 10–15 repetitions, ~80% 1RM, 3d per week) RET programme (Van Roie et al., 2013). The present study adds to the current body of evidence on muscle activity during stair climbing by characterising both the descending and ascending loading component. Specifically, we observed lower quadriceps muscle EMG activity during the descending loading pattern (Young, ~4%, Old, ~14%) compared with the stair ascent (Young, ~6%, Old, ~20%). The lower EMG activity during the descent may be partially explained by the heightened antagonistic muscle pre-activation and co-activation of biceps femoris and tibialis anterior associated with age-related leg stiffness (Hortobágyi and DeVita, 2000). Nevertheless, it is pertinent to note that descending stair walking has recently been observed to be superior to ascending stair walking following a twelve week training intervention on markers of metabolic health (Honda et al., 2016), quadriceps isometric strength and functional fitness (Chen et al., 2017) in older adults with metabolic syndrome. Whilst the evidence regarding stair climbing as a potential exercise stimulus certainly lacks substantial acute and longitudinal physiological evidence, the current body of literature suggests that stair climbing should be incorporated habitually into ADL to help support the maintenance of muscle mass in older age. Moving forward, it would be prudent to understand the long-term muscle-specific and whole-body metabolic benefits of stair climbing exercise in older individuals when compared directly with other accessible exercise modes (i.e. chair-based sit-to-stand and elastic bands).

Whilst textile embedded EMG shorts has previously been used in a variety of experimental settings (Bengs et al., 2017; Tikkanen et al., 2013; Tikkanen et al., 2016), we acknowledge that the sensitivity of this surface EMG technique does not allow us to explore complex neuromuscular patterns, compared with methods such as the application of indwelling intramuscular (Piasecki et al., 2016; Piasecki et al., 2018b) and high-density EMG (Martinez-Valdes et al., 2016), nor does it allow us to determine the EMG activity of the whole quadriceps muscle. The normalization of muscle EMG activity was conducted during knee extensor MVC at a 90^o^ knee angle, comparable to the starting position of MN-RET and EB-RET. Whilst it has long been disputed whether to normalize EMG to isometric (Halaki and Ginn, 2012), dynamic-isokinetic (Burden and Bartlett, 1999) and/or during locomotion (Chuang and Acker, 2019), it is pertinent to note that the most appropriate method of EMG normalization is lacking consensus (Halaki and Ginn, 2012). The present investigation also utilised an acute within-subject, within-muscle approach to compare and contrast muscle EMG activity across a range of activities and exercise modes to account for inter-individual variability and heterogeneity (Vigotsky et al., 2018). However, despite similar investigations finding no differences between EB and MN-RET (Jakobsen et al., 2013; Jakobsen et al., 2012; Jakobsen et al., 2019), we are not able to infer whether EB-RET would be superior at supporting longitudinal gains in skeletal muscle mass and strength compared with machine-based alternatives.

In conclusion, the present study suggests that performing EB-RET, but not BW-RET, elicits similar increases in quadriceps muscle EMG activity to that of traditional machine-based resistance exercise in both younger and older adults. Conversely, precise exercise prescription, either by training to volitional failure, or adding external load may be necessary to augment quadriceps specific muscle activity in BW-RET in older adults. Moreover, EB and BW modes of RET do not require specialised equipment and/or facilities, and therefore, may be prescribed alone and/or in combination as a pragmatic and feasible home-based RET alternative to traditional machines for older adults to maintain and improve skeletal muscle mass, strength and function. Finally, it is apparent that the declines in muscle mass and strength associated with ageing significantly increase the relative muscular effort of ADL and RET in older adults.

## Funding

This work was funded by an MRC-ARUK Centre for Musculoskeletal Ageing Research studentship to RNM.

## Author contributions

RNM, PTM, EMV and LB conceived and designed the research. RNM and PTM conducted all experiments. EMV provided assistance with data analysis. RNM and PTM wrote the manuscript. PTM, EMV and LB supervised the project throughout. All authors contributed to the interpretation of results and read, edited and approved the manuscript.

## Declaration of competing statement

The authors declare that the research was conducted in the absence of any commercial or financial relationships that could be construed as a potential conflict of interest.

## Appendix A Appendix A Supplementary data

Supplementary figures Image 1
