# Supplementary material for: Quadriceps muscle electromyography activity during physical activities and resistance exercise modes in younger and older adults
Source: Exp Gerontol. 2020 Jul 15;136:110965. doi: 10.1016/j.exger.2020.110965 (PMC7264709; doi:10.1016/j.exger.2020.110965)
Supplement: Supplementary file 1 — Supplementary figures [file mmc1.docx]

**Supplementary Material**

**Figure 4. Example of Participant Undertaking Functional Exercise Tasks.**

Images from left to right: 15 m Walk (A, B, C), Stair Assent (D, E, F), Stair Descent (G, H, I)


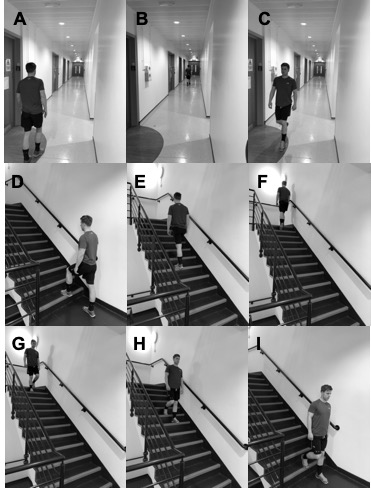


**Figure 5. Example of Resistance Exercise Modes**

Start (A, C and E) and end position (B, D and F) for bodyweight chair squat (A, B), elastic band knee extension exercise (C, D), machine knee extension exercise (E, F), respectively.


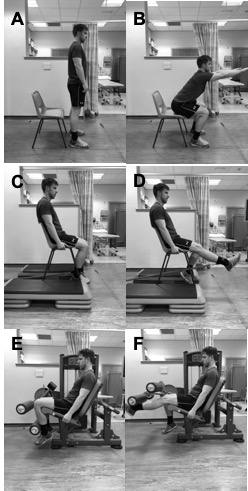


**Figure 6. Image of Textile EMG shorts.**

Textile-embedded EMG shorts from front (A) and inside Out (B) perspective.


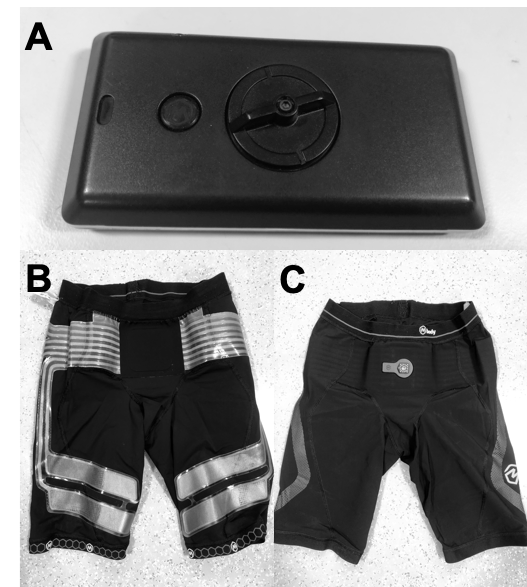


**Figure 7: Example of Filtered 25 Hz EMG Trace of Activities of Daily Living and Resistance Exercise Modes for Younger and Older Adults.**

Images from left to right: Maximal Voluntary Contraction (A), Walking (B), Stair Climb (C), Bodyweight Chair Squat (D), Elastic Band Resistance Exercise (E), Machine Resistance Exercise. Blue line and red line indicated young and old respectively.
